# Cerebral Malaria in Mouse and Man

**DOI:** 10.3389/fimmu.2018.02016

**Published:** 2018-09-10

**Authors:** Nazanin Ghazanfari, Scott N. Mueller, William R. Heath

**Affiliations:** ^1^Department of Microbiology and Immunology, The Peter Doherty Institute for Infection and Immunity, University of Melbourne, Melbourne, VIC, Australia; ^2^The ARC Centre of Excellence in Advanced Molecular Imaging, University of Melbourne, Melbourne, VIC, Australia

**Keywords:** cerebral malaria, *Plasmodium falciparum*, *Plasmodium berghei*, blood-brain barrier, T cells

## Abstract

Cerebral malaria (CM) is an acute encephalopathy caused by the malaria parasite *Plasmodium falciparum*, which develops in a small minority of infected patients and is responsible for the majority of deaths in African children. Despite decades of research on CM, the pathogenic mechanisms are still relatively poorly defined. Nevertheless, many studies in recent years, using a combination of animal models, *in vitro* cell culture work, and human patients, provide significant insight into the pathologic mechanisms leading to CM. In this review, we summarize recent findings from mouse models and human studies on the pathogenesis of CM, understanding of which may enable development of novel therapeutic approaches.

## Introduction

Malaria is a life-threatening disease with an estimated 216 million cases of disease and ~445,000 deaths in 2016 (1). The majority of cases of malaria are among children under the age of five and pregnant women in sub-Saharan Africa. Human malaria is caused by five different species of *Plasmodium* parasites, of which *Plasmodium falciparum* and *Plasmodium vivax* are the most prevalent and *Plasmodium falciparum* is the most lethal. Human infections can also be caused by *Plasmodium malariae, Plasmodium ovale*, and *Plasmodium knowlesi* (2). Female Anopheles mosquitoes transmit malaria by injecting sporozoites into the host while taking a blood meal (3). These parasites travel to the liver and invade hepatocytes and then multiply and develop into schizonts. After about a week, the hepatic schizonts burst and release thousands of merozoites that invade erythrocytes. Within the erythrocyte, the merozoites begin the asexual cycle, which takes approximately 24 h for *P. knowlesi*, 48 h for *P. falciparum, P. vivax*, and *P. ovale*, and 72 h for *P. malariae* (4). The erythrocytic schizont then ruptures and releases merozoites that can invade erythrocytes and repeat the erythrocytic cycle. Some of the merozoites also develop into gametocytes that can transmit malaria to mosquitoes (3). The liver-stage of malaria is asymptomatic, with disease symptoms all deriving from the cycling of parasites in the blood (5, 6). The clinical presentation of malaria differs depending on the age of the patient and whether they have had previous exposure (7). Fully immune individuals in malaria endemic areas will largely endure asymptomatic malaria. Most patients suffer from uncomplicated malaria with mild symptoms such as fever, headache, chills and vomiting. Some patients with *Plasmodium falciparum* malaria develop severe complications like severe anemia, respiratory complications and acidosis or cerebral malaria. In adults, multi-organ failure is also frequent (8–10).

## Cerebral malaria

Cerebral malaria (CM) is a severe neurological complication of infection with *Plasmodium falciparum*. It causes a diffuse encephalopathy associated with coma and is responsible for most malaria-related deaths globally. CM is defined by the WHO as a clinical syndrome characterized by coma with the presence of asexual forms of *Plasmodium falciparum* in peripheral blood, and exclusion of other factors that could cause unconsciousness such as other infections or hypoglycemia (2). CM causes 15–20% mortality, despite effective antimalarial therapy and intensive care, and survivors may develop long-term neurological deficits (11–13). This severe form of the disease is most frequent in sub-Saharan Africa, where malaria transmission is intense. In this region, CM principally occurs in children under five and is rare in adults. However, in South East Asia, where malaria transmission is low, CM principally occurs in adults (2). The clinical manifestations of CM differ between children and adults (14–16), suggesting that different pathophysiological features are associated with human cerebral malaria (HCM) depending on age. In adults, CM is often accompanied by multi-organ complications, including central nervous system dysfunction, liver dysfunction, acute kidney failure, and respiratory failure. In contrast, in African children, CM usually manifests as coma, seizures, and severe anemia, but respiratory and renal failure are generally rare (14, 17, 18). Retinal abnormalities including retinal hemorrhages, papilledema, retinal whitening and retinal vessel color changes are common in children with CM. Components of retinopathy can be useful for distinguishing malarial from non-malarial coma (19–23).

## Pathogenesis of CM

The precise mechanisms involved in the pathogenesis of HCM are not fully understood. Most observations regarding HCM have relied on examination of post-mortem samples. Sequestration of infected red blood cells (iRBC) in the brain of *P. falciparum*-infected people is a hallmark of CM in adults (24–27) and children (23), and has been proposed as the main process responsible for HCM development (28). The iRBCs that contain mature parasites disappear from the peripheral circulation and specifically localize in microvessels of the brain and other organs. Adhesion of iRBCs to vascular endothelium is mediated by *Plasmodium falciparum* erythrocyte membrane protein-1 (PfEMP-1), a specific cell-surface ligand expressed by iRBCs. PfEMP-1 is able to bind to many host ligands on endothelial cells, such as CD36, the intercellular adhesion molecule 1 (ICAM-1) and the endothelial protein C receptor (EPCR) (29–32). It has been suggested that accumulation of iRBCs in the cerebral capillaries causes mechanical obstruction of the vessels leading to a reduction in blood flow, hypoxia, coma, and death (24). Many studies have reported that there is a significant correlation between sequestration of iRBCs in cerebral vessels and coma in patients with CM (24–27, 33). However, sequestration of iRBCs in the brain capillaries might not be essential to cause CM, because some patients clinically diagnosed with HCM had little or no sequestration of iRBCs in their brain (23). Some of these latter patients were clearly a consequence of misdiagnosis (23), supporting the view that parasite sequestration is essential for HCM.

## A rodent model of experimental CM

A well-characterized model of experimental cerebral malaria (ECM), which utilizes *P. berghei* ANKA (PbA) infection of various mouse strains including C57BL/6, has been widely employed to dissect the mechanisms involved in CM. This ECM model shares some similarities with *P. falciparum* HCM (34). Here, susceptible mice infected with PbA develop neurological signs such as paralysis, ataxia, convulsion, and/or coma and die within the first 2 weeks of infection (35). However, in murine CM, sequestration of iRBC in the brain vasculature is not a major histopathologic feature. While some studies have reported that PbA iRBC can accumulate in different organs, including the murine brain (36, 37), a correlation between iRBC sequestration and ECM was not demonstrated (36, 37). Nevertheless, one study showed that accumulation of iRBC in the brain of mice is crucial for the development of ECM (38). Other studies used luciferase-expressing PbA to report that iRBC sequestration in various organs, including the brain, was associated with the onset of ECM (39, 40). They observed a rapid increase in parasite biomass in various organs of infected mice at the time mice developed clinical signs. More recently, Strangward et al. showed accumulation of iRBCs in brain blood vessels was a specific feature of ECM caused by PbA and was not observed during uncomplicated *P. berghei* NK65 infection (41). This study suggested that a single iRBC is sufficient to occlude a brain capillary in PbA-infected mice, implicating this process as a contributing attribute in the pathogenesis of ECM, thus paralleling human CM.

## The role of immune cells

Immune system effector cells have been proposed to be involved in the pathogeneses of CM. In addition to iRBCs, some studies have reported host cells such as leukocytes and platelets within brain microvessels of patients with CM (42–45), though this observation is not universal (24, 27). Grau et al. showed that platelet accumulation occurs in the cerebral microvasculature of Malawian children with CM and that platelets are colocalized with malaria pigment and white cells in most patients with CM (42). They have also reported that platelet accumulation in brain microvessels were significantly higher in patients with CM than in those with either severe malarial anemia or nonmalarial encephalopathies. Another autopsy study reported intravascular accumulations of monocytes and platelets in the brain microvasculature of children with fatal CM (45). This study also showed that children with autopsy-confirmed CM had significantly more brain intravascular monocytes and platelets than did children with other causes of death. In contrast, studies examining the brains of adult patients from Thailand, observed little or no accumulation of leukocytes or platelets within brain tissue (24, 27). Intravascular accumulation of immune cells has been extensively observed in the brains of mice with ECM (46–48). These immune cells are mainly composed of T cells, neutrophils, monocytes, and natural killer (NK) cells. It has been shown that the migration of leukocytes to the brain occurs at the same time as the neurological signs of ECM appear. NK cells have been reported to be required for the development of ECM and are recruited to the brain of PbA-infected C57BL/6 mice (47). Here, depletion of NK cells using anti-asialo-GM1 antibody protected mice from CM by inhibiting the migration of CD8^+^ and CD4^+^ T cells to the brain. This result should be taken with some caution, however, since activated CD8^+^ T cells can also express this marker and are essential for ECM.

The involvement of monocytes, macrophages and neutrophils in the pathogenesis of ECM is still unclear. Antibody depletion of neutrophils and macrophages shortly before manifestation of neurological signs did not prevent the development of CM in PbA-infected mice, suggesting that these cells are not involved in the effector phase of ECM (46). Pai et al. have shown that depletion of monocytes prior to PbA infection resulted in complete protection from ECM (49). However, depletion of monocytes late after PbA infection had no effect, indicating that monocytes are not essential to ECM pathology. Nevertheless, these mice showed a significant reduction in the number of CD8^+^ T cells, CD4^+^ T cells, and NK cells within the brain, suggesting that monocytes/macrophages play a limited role in the recruitment of lymphocytes to the brain during ECM. This limited role was further emphasized in a recent study that depleted neutrophils with anti-Ly6G antibodies in CCR2^−/−^ mice (which also lack circulating monocytes) prior to PbA infection and showed this did not prevent ECM development (50).

Platelets have been implicated in ECM based on evidence of accumulation within brain microvessels of diseased mice (51). Intravital imaging of the brain of mice with ECM revealed small clusters of platelets were marginalized in post-capillary venules and that these clusters were co-localized with patches of P-selectin (52, 53). These observations may be important, as depletion of platelets by anti-CD41 mAb in early but not late stages of ECM development protected mice from disease, implicating platelets in ECM pathology (54, 55). Activation of platelets leads to the release of large amounts of CD40 ligand (CD40L, CD154), which may interact with CD40 constitutively expressed on endothelial cells (56, 57). Mortality and breakdown of the blood-brain barrier (BBB) were prevented in PbA-infected mice lacking either CD40 or CD40L (58). Macrophage sequestration was also reduced in brain vessels of these mice. While these data may implicate platelets and platelet-derived CD40L in ECM, caution should be taken in this interpretation as CD40 has other important roles in ECM, such as in the provision of CD4^+^ T cell help for the generation of parasite-specific CD8^+^ T cell responses (59). Supporting a role for platelet-derived CD40L, it has been shown that adoptive transfer of WT CD40^+^ platelets into CD40-deficient mice, increased ECM mortality (60).

Accumulation of both CD8^+^ T cells and CD4^+^ T cells in the brain of PbA-infected mice has been described in several studies (46–48, 61, 62). A number of these and other reports have also demonstrated that CD8^+^ T cells are required for the development of ECM. Antibody depletion of CD8^+^ T cells in PbA-infected F_1_ (129/Ola × C57BL/6J) and 129P2Sv/ev mice shortly before manifestation of neurological signs prevented the development of ECM (46, 48). Depletion of CD8^+^ T cells in PbA-infected B6 mice 4 days after infection prevented the vascular hemorrhaging, BBB breakdown, and the development of ECM (50). CD8-deficient (40, 46, 63) or β2-microglobulin-deficient mice, which lack functional CD8^+^ T cells (64) also failed to develop ECM. These results suggest that brain-sequestered CD8^+^ T cells play an important effector role in the development of ECM. This was confirmed by adoptive transfer experiments where splenic CD8^+^ T cells or CD8^−^ T cells from PbA-infected C57BL6 mice were transferred into ECM-resistant, RAG2-KO recipient mice that were subsequently infected with PbA (61). RAG2-KO recipient mice developed ECM after transfer of CD8^+^ T cells, but not after transfer of CD4^+^ T cells, suggesting that cytotoxic CD8^+^ T cells are critical for the development of ECM. In addition, adoptive transfer of CD8^+^ T cells isolated from perforin-deficient mice into RAG2-KO recipient mice did not cause ECM (61). These results suggest that perforin-dependent cytotoxic pathways are involved in the pathogenesis of ECM. It has also been shown that expression of granzyme B by CD8^+^ T cells is essential for the development of ECM (65). Granzyme B-deficient mice had significantly lower total parasite burdens in the brain and were completely resistant to ECM. However, similar numbers of infiltrating CD8^+^ T cells were found in the brains of PbA-infected wild-type and granzyme B-deficient mice (65), suggesting granzyme B was required in the effector phase rather than in development or sequestration of CD8^+^ T cells to the brain. CD8^+^ T cells have been shown to promote iRBC accumulation in the brain and other organs during ECM (39, 40). It has been shown that significantly fewer iRBC accumulated in brains, spleens and other organs of CD8^+^ T cell-depleted mice than in WT or CD4^+^ T cell-depleted mice (40). Baptista et al. have shown that depletion of CD8^+^ T cells a day prior to the onset of ECM protected PbA-infected mice and reduced the accumulation of iRBC in the brains of those mice (38). They have also shown that treatment with antimalarial drugs delayed development of ECM in PbA-infected mice without altering the number of CD8^+^ T cells in the brain (38). These results suggest that the brain sequestration of CD8^+^ T cells is not sufficient for the development of ECM and that the presence of both CD8^+^ T cells and iRBC in the brain is crucial. Baptista et al. (38), also reported the accumulation of CD8^+^ T cells in the brains of mice infected with *P.berghei* NK65 (a parasite line that does not cause ECM). However, neither accumulation of iRBCs in the brain nor BBB breakdown was observed in these mice, suggesting iRBC accumulation is important for the onset of ECM. The requirement of iRBC accumulation may underpin a need for presentation of parasite antigens by MHC I molecules on brain endothelium (50). Parasite-specific T cells were shown to spend greater time than non-specific T cells arrested on brain blood vessels of mice undergoing ECM and this interaction could be impaired by antibody that blocked recognition of MHC I-peptide complexes. Abrogation of ECM in chimeric mice where brain endothelium lacked expression of MHC I molecules further supports this view.

It has been shown that CD4^+^ T cells can either play a role in the induction phase or in both the induction and in the effector phase of ECM. Antibody depletion of CD4^+^ T cells in PbA-infected F_1_ (129/Ola X C57BL/6J) (46), CBA/Ca (66), and C57BL/6 mice (39, 64) prevented the development of ECM when conducted before or early in the infection, suggesting an essential role for CD4^+^ T cells in the initiation of ECM pathogenesis. A role for CD4^+^ T cells in ECM pathogenesis was also supported by experiments where PbA infection of CD4-deficient mice did not lead to the development of ECM (46, 63, 64). A recent study has reported that depletion of CD4^+^ T cells 4 days after infection did not protect PbA-infected B6 mice from ECM (50). Given the importance of CD8^+^ T cells in ECM pathogenesis and the well-established role for CD4^+^ T cells in helping CD8^+^ T cells in many infection models (67), the requirement for CD4^+^ T cells in ECM pathogenesis may reflect a similar helper role. This view is strongly supported by the capacity of wild-type PbA-specific CD4^+^ T cells to provide help for CD8^+^ T cell expansion and ECM development in CD40 ligand-deficient mice, where endogenous CD4^+^ T cells lack expression of this receptor (59). The role of CD4^+^ T cells in the effector phase of ECM is still unclear. In some studies, using C57BL/6 or (129/Ola x C57BL/6) F_1_ mice, depletion of CD4^+^ T cells immediately before the onset of neurological signs did not prevent development of ECM (40, 46). However, for 129P2Sv/ev mice, depletion of CD4^+^ T cells late after PbA infection prevented the development of ECM (48). In another study, the majority of C57BL/6 and C57B1/10 mice infected with PbA were protected from ECM development when CD4^+^ T cell depletion was undertaken just prior to normal disease onset (68). Thus, while all studies agree with a requirement for CD4^+^ T cells, whether this is largely during the priming phase or in part contributed in the effector phase may depend on the strain of mice and perhaps the environment where experiments are performed.

## The role of cytokines, chemokines, and adhesion molecules

Pro-inflammatory cytokines such as lymphotoxin α (69) and IFN-γ (70, 71) have been implicated in the development of ECM during PbA infection. Elevated blood concentrations of cytokines, particularly TNF and IFN-γ were found in patients with CM (72–76) and also in animal models of ECM (71, 77). An essential role for TNF has been excluded for ECM, however, since TNF-deficient mice are susceptible to PbA-induced disease (69). The key role of IFN-γ in the pathogenesis of ECM was confirmed by experiments where PbA-infected IFN-γ^−/−^ and IFN-γ receptor (IFN-γR)^−/−^ mice did not develop ECM, nor show parasite or leukocyte accumulation in their brains (48, 71). IFN-γ was shown to be involved in the control of iRBC accumulation in the brain and other organs of PbA-infected mice (39, 40). IFN-γ also promotes the up-regulation of adhesion molecules such as ICAM-1 on brain endothelial cells during malaria infection (71, 78). Cerebral endothelial cells from PbA-infected IFN-γ^−/−^ mice showed significantly reduced expression of adhesion and antigen presenting molecules when compared to wild type mice (50). It has been shown that IFN-γ can be produced in response to *Plasmodium* parasites by many cell types, including NKT cells, NK cells, γδ TCR^+^ T cells, and αβ TCR^+^ CD4^+^ and CD8^+^ T cells (79). Adoptive transfer of infection-derived CD4^+^ T cells, but not innate or CD8^+^ T cells, into normally resistant IFN-γ^−/−^ mice (infected with PbA) promoted the development of ECM by active secretion of IFN-γ, implicating cytokine derived from CD4^+^ T cells in ECM (80). This conclusion was further supported by showing that IFN-γ-producing CD4^+^ T cells enhanced the expression of CXCL9 (Mig) and CXCL10 (IP-10) and induced CD8^+^ T cell migration and accumulation within the brain of PbA-infected IFN-γ^−/−^ mice.

Endothelial cell adhesion molecules are believed to play an important role in the pathogenesis of cerebral malaria. Elevated circulating levels of pro-inflammatory cytokines including IFN-γ and TNF during malaria infection result in an intense up-regulation of endothelial cell adhesion molecules such as ICAM-1, VCAM-1, and P-selectin in PbA-infected mice (78). Marked up-regulation of ICAM-1, VCAM-1, and E-selectin on brain endothelial cells has also been observed in patients with CM (81–83). Postmortem studies have shown that there is co-localization between sequestered iRBCs and ICAM-1 in the cerebral vessels (82). The importance of ICAM-I in the development of ECM was confirmed by experiments where PbA-infected ICAM-1^−/−^ mice did not develop ECM (84). This was associated with a slight reduction in macrophage sequestration to the brain and an absence of BBB breakdown (84). In another study (50), late treatment of PbA-infected mice with a combination of anti-LFA-1 and anti-VLA-4 antibodies was shown to disrupt the CD8^+^ T cell interactions with brain endothelial cell expressed ICAM-1 and VCAM-1, resulting in a rapid displacement of PbA-specific CD8^+^ T cells from the cerebral vessels, protecting mice from ECM.

CXCR3, a T cell chemokine receptor, has been shown to be necessary for recruitment of T cells into the brain and the development of ECM (85). A majority of PbA-infected CXCR3-deficient mice did not develop ECM and showed reduced numbers of CD8^+^ T cells in their brain vessels (54, 62, 85). Enhanced expression of CXCR3-binding chemokines such as CXCL9 and CXCL10 has been reported in the brains of PbA-infected mice and mice deficient in either chemokine were partially protected from ECM (85). Mice lacking CXCL10 were also shown to have increased retention of T cells in the spleen and reduced T cell infiltration in the brain, coinciding with reduced disease (86). A recent study has also reported that CXCL10 produced by brain endothelial cells induces the adhesion of T cells to cerebrovascular endothelium and prevents T cell detachment from the brain vasculature in PbA-infected mice. The induction of CXCL10 was shown to be IFN-γ dependent (87).

CXCL4, which is also a ligand for the chemokine receptor CXCR3, is released by activated platelets early in the course of ECM (54). CXCL4 has been shown to be necessary for the development of ECM, as the majority of mice deficient in CXCL4 are protected from disease (54). It was shown that CXCL4 stimulates the production of TNF by T cells and macrophages as well as inducing T cell migration to the brain.

## The blood-brain barrier (BBB) in CM

The BBB is an interface between the intravascular space and the central nervous system (CNS) that regulates passage of molecules from the blood into the brain and the transport of carbon dioxide and metabolic waste products from the brain into the blood (88). There are two functionally distinct blood brain barriers (89); (i) a physiological BBB that regulates diffusion of solutes and is formed by capillaries - it encompasses a single layer composed of endothelial cells, gliovascular membrane, and astrocyte endfeet of the glia limitans, and (ii) a neuroimmunological BBB, which is formed by post-capillary venules and consists of two layers, the vascular endothelium with its basement membrane and the glia limitans with associated astrocyte endfeet and their basement membrane. These two layers are separated by perivascular space. In an inflammatory response, immune cells migrate into the CNS at the neuroimmunological BBB. Here, immune cells need to cross two physical barriers, the vascular endothelium and the glia limitans to enter the CNS parenchyma (89, 90). Whether lymphocyte crossing of the BBB is essential for development of ECM is unclear. Evidence that blocking the adhesion molecules LFA-1 and VLA-4 to release CD8^+^ T cells accumulating within the lumen of blood vessels prevents ECM, yet has no effect on CD8^+^ T cells within the brain parenchyma (50), suggests those cells that cross the BBB are not essential for disease. What appears to be more important is breakdown of solute exclusion by the BBB and consequent swelling of the brain.

Many studies have examined BBB alterations in *P. falciparum* patients by measuring the level of molecules such as albumin or immunoglobulins (IgG) in the cerebrospinal fluid (CSF) and plasma. Albumin is not synthesized intrathecally and is excluded from the brain by an intact BBB whereas IgG can be synthesized intrathecally by plasma cells. Calculation of the albumin index, [albumin] _CSF_/[albumin]_plasma_, is used to examine BBB integrity in individuals. Calculation of the IgG index, ([IgG] _CSF_ X [albumin] _plasma_/[IgG] _plasma_ X (albumin) _CSF_) is a traditional method of detecting intrathecal IgG production (91). It has been shown that radioactive ^125^I-labeled albumin levels in the CSF of Thai adult patients with CM were not increased after injection during and after coma (92). In another study, albumin and IgG levels in the CSF of Vietnamese adult patients with CM were not elevated compared with control subjects, except in a few individual cases (93). In contrast, Malawian children with CM showed higher levels of albumin in the CSF compared with UK adult controls, although no difference was found in CSF albumin levels between children who died vs. those who survived from the disease (94). Measuring the local synthesis of IgG within the CNS showed an increased IgG index in the CSF of 43% of adult Thai patients with CM (95). All together, these studies suggest that some degree of permeabilisation of the BBB occurs in individual patients with CM.

Postmortem studies of the brains of Malawian children who died from *P. falciparum* malaria have shown the presence of myelin and axonal damage, BBB breakdown, and glial responses, in addition to the sequestration of iRBCs in brain microvessels (44). Axonal damage, demyelination, and ring hemorrhages have also been observed in the postmortem brain tissue of Vietnamese adult patients who died from *P. falciparum* malaria (96). Another recent study directly compared retinal and cerebral histopathological changes in the same patients who died from CM. They found similar pathological features including hemorrhages, sequestration of iRBCs, axonal damage, and BBB disruption in the retina and the brain of individual patients with fatal CM (97). The alteration of the BBB in mice with ECM was confirmed by measuring the movement of the dye Evans Blue, radio-labeled albumin, radio-labeled antibody or horseradish peroxidase (52, 98, 99). Several pathologic changes including brain edema, enlarged perivascular spaces, BBB breakdown, and vascular leakage have been observed in mice with ECM (52, 100, 101). ECM studies have also revealed evidence of cell death as well as vascular leakage for multiple brain regions, particularly in the brainstem and olfactory bulb (50). In this study, the majority of the dead cells in the brainstem were shown to be neurons. Cell death within the brainstem of mice undergoing ECM is likely caused by brain swelling, which parallels observations in CM patients, where brain swelling caused by edema leads to lethal cerebral herniation causing damage to this region (102).

The exact mechanisms responsible for the BBB alterations are not fully characterized. A reduction in expression of the endothelial tight junction–associated proteins, which are important for maintaining BBB integrity, has been observed in vessels of human brains, where they colocalize with areas of sequestered iRBCs (94, 103). A reduction in expression of endothelial tight junction proteins in areas of vascular breakdown in the brain has also been observed for mice undergoing ECM (50). These data suggest modulation of tight-junction-associated protein expression may contribute to BBB alterations, possibly by disrupting the connections that maintain BBB integrity.

*In vitro* studies have shown that the capture of iRBC-associated material by brain endothelial cells leads to the opening of intercellular tight junctions (104). Capture of parasite material *in vivo* has also been shown to enable brain endothelial cells to cross-present PbA antigens on MHC I, in an IFN-γ-dependent manner (105). Recognition of these antigens by CD8^+^ T cells *in vivo* also affects tight junction integrity (50). Monocyte adherence to endothelial cells in the retinal vessels is also accompanied by an increase in BBB permeability (106, 107), potentially implicating these cells in this process. Platelet adhesion to brain endothelial cells also seems to contribute to alteration of the BBB. It has been shown that platelets can act as a bridge between iRBC and the surface of brain endothelial cells and may therefore promote the adhesion of iRBC to the endothelial vascular (108). Low plasma platelet counts and binding of iRBC to EPCR by PfEMP1 has been linked to brain swelling and retinopathy in CM patients, suggesting that binding of platelets may precipitate or contribute to endothelial disruption and edema (109). Platelets were also found to potentiate apoptosis of TNF-stimulated human brain microvascular endothelial cells through TGF-β *in vitro* (110). EPCR binding by iRBC has also been implicated in BBB breakdown through a local reduction in the generation of activated protein C causing down-stream effects on protease activated receptor 1 (PAR1) that alter vascular permeability (111).

Matrix metalloproteinases are zinc-dependent endopeptidases that are involved in many aspects of immunity (112). These enzymes can degrade extracellular matrix proteins as well as non-matrix targets, such as secreted cytokines, chemokines, and cell surface receptors (113). Elevated MMP expression or activity has been implicated in many disease processes (114). It has been shown that MMP-9 is crucial for the disruption of the BBB in several CNS diseases (115, 116). MMP-2 and MMP-9 activity at the parenchymal border are crucial for infiltration of leukocytes into the brain parenchyma in a mouse experimental autoimmune encephalomyelitis (EAE) model (117). MMP-2 and MMP-9 double knockout mice are resistant to EAE and leukocyte infiltration into the CNS is prevented in these mice (117). Elevated levels of several MMPs have been reported during ECM in different organs and inhibition of such MMPs with BB-94, a broad-spectrum MMP-inhibitor, delayed the onset of ECM by 1 day (118). There was a significant increase in the expression of activated forms of MMP-9 in the brain of PbA-infected C57/BL6 mice late in disease. However, no significant differences in lethality were observed between MMP-9 knockout mice and wild type mice during the course of ECM, suggesting that this MMP does not play a key role in the pathogenesis of ECM.

## Imaging approaches for investigating the pathogenesis of CM

Several imaging techniques have been used to investigate the pathogenesis of cerebral malaria in living subjects. Computed tomography (CT) scans and magnetic resonance imaging (MRI) have provided some insights into human CM pathogenesis. Acute head CTs revealed some of the pathological changes in the brains of children with retinopathy-confirmed cerebral malaria including large vessel infarcts, edema, and herniation. Follow-up CT scans in survivors identified focal cortical atrophy that correlated with regions affected by focal seizures during acute cerebral malaria (119). A large MRI study was performed in Malawian children with CM and compared findings in unconscious patients with and without retinopathy. MRI findings revealed a wide range of abnormalities including brain swelling and severe edema, which were more common in patients with malaria retinopathy (120). In another MRI study, African children who met the definition of cerebral malaria and had retinopathy underwent MRI examination (102). MRI results showed that the majority (84%) of children who died from CM had severe brain swelling at admission. In contrast, evidence of severe brain swelling was only observed in 27% of survivors, and in these cases temporal MRI imaging showed that swelling was transient.

In a recent study, MRI was performed in adult Bangladeshi patients with severe *falciparum* malaria (121). Diffuse mild brain swelling, mostly without edema, was a common abnormality observed in patients with severe *falciparum* malaria and was not specific to patients with coma or fatal disease. The majority of patients had malarial retinopathy. Retinal changes were more severe and common in patients with coma (121). While these approaches provide valuable information regarding the pathological changes in the brains of malaria patients, they are limited in their capacity to investigate pathogenic mechanisms at a cellular and molecular level.

In mice, ultra-high-field MRI was used to identify the olfactory bulb as a vulnerable part of the brain during PbA infection (122). MRI images showed that micro-hemorrhages occur in the olfactory bulb when ECM symptoms begin, prior to other parts of the brain showing clear evidence of bleeding. Whole animal imaging is a technique that enables the visualization of parasite load in different organs of intact malaria-infected animals by using luciferase expressing parasites. While this approach is not suitable for monitoring individual parasites or immune cells, it has been of value to show a rapid increase in parasite biomass in the brains of mice at ECM onset (38–40).

Intravital microscopy (IVM) enables the visualization of individual cell interactions in live animals and is a useful tool for investigating the pathogenesis of ECM. A recent IVM study has suggested that ECM correlates with widespread opening of the neuroimmunological BBB and that this occurs without widespread loss of vascular endothelial cells in the brain (52). This study shows that mice with ECM, but not hyperparasitemia, exhibit leukocyte arrest, CD14 expression, platelet marginalization, and vascular leakage from post-capillary venules, but not capillaries or arterioles. Another IVM study from the same group reported the accumulation of numerous CD8^+^ T cells, neutrophils, and macrophages within post-capillary venules of mice with ECM (53). Others showed that monocytes start to accumulate in the brain blood vessels 1–2 days prior to the onset of ECM and that activated CD8^+^ T cells regulate monocyte accumulation in mice during ECM (49). As the disease progressed in PbA-infected mice, a significant reduction was observed in rolling velocity of monocytes, which was accompanied by a significant increase in the number of monocytes adhering to the microvasculature of the brain (49). Examination of the location of CD8^+^ T cells within the brain revealed that the majority of infiltrating T cells accumulate on the perivascular side of the blood vessels, but this was only seen in mice infected with PbA but not Pb NK65 parasites (123). The accumulating CD8^+^ T cells within the brains of mice infected with both Pb ANKA and Pb NK65 were similarly activated, but they exhibited different movement characteristics. This study also showed that infected red blood cells accumulated both in intravascular and perivascular spaces at the time of ECM development in PbA-infected mice. Significantly less parasite accumulation was observed in the brains of mice infected with Pb NK65 potentially explaining the lack of ECM in this model.

Swanson et al. reported that PbA-specific CD8^+^ T cells arrested along both the luminal and extravascular surfaces of cerebral vasculature at the peak of ECM, although the majority interacted on the luminal side (50). They found that the arrest of PbA-specific CD8^+^ T cells was specific for brain vasculature and was not observed in other peripheral tissues. iRBCs were also observed on both the luminal and abluminal surfaces of cerebral blood vessels of these mice and were actively phagocytosed by myelomonocytic cells and perivascular macrophages. Importantly, interactions of parasite-specific CD8^+^ T cells with the luminal side of brain blood vessels was impaired by blocking interactions with MHC I molecules, which together with the lack of ECM development in chimeric mice where MHC I was not present on endothelial cells or where T cell adhesion was inhibited by blocking the integrins VLA-4 and LFA-1, strongly implicated cognate interactions of CD8^+^ T cells with brain endothelium in the process of ECM development. Together, intravital imaging studies highlight the contribution of sequestered iRBCs and brain-infiltrating immune cells to the pathogenesis of ECM.

## Summary

**Figure 1:**
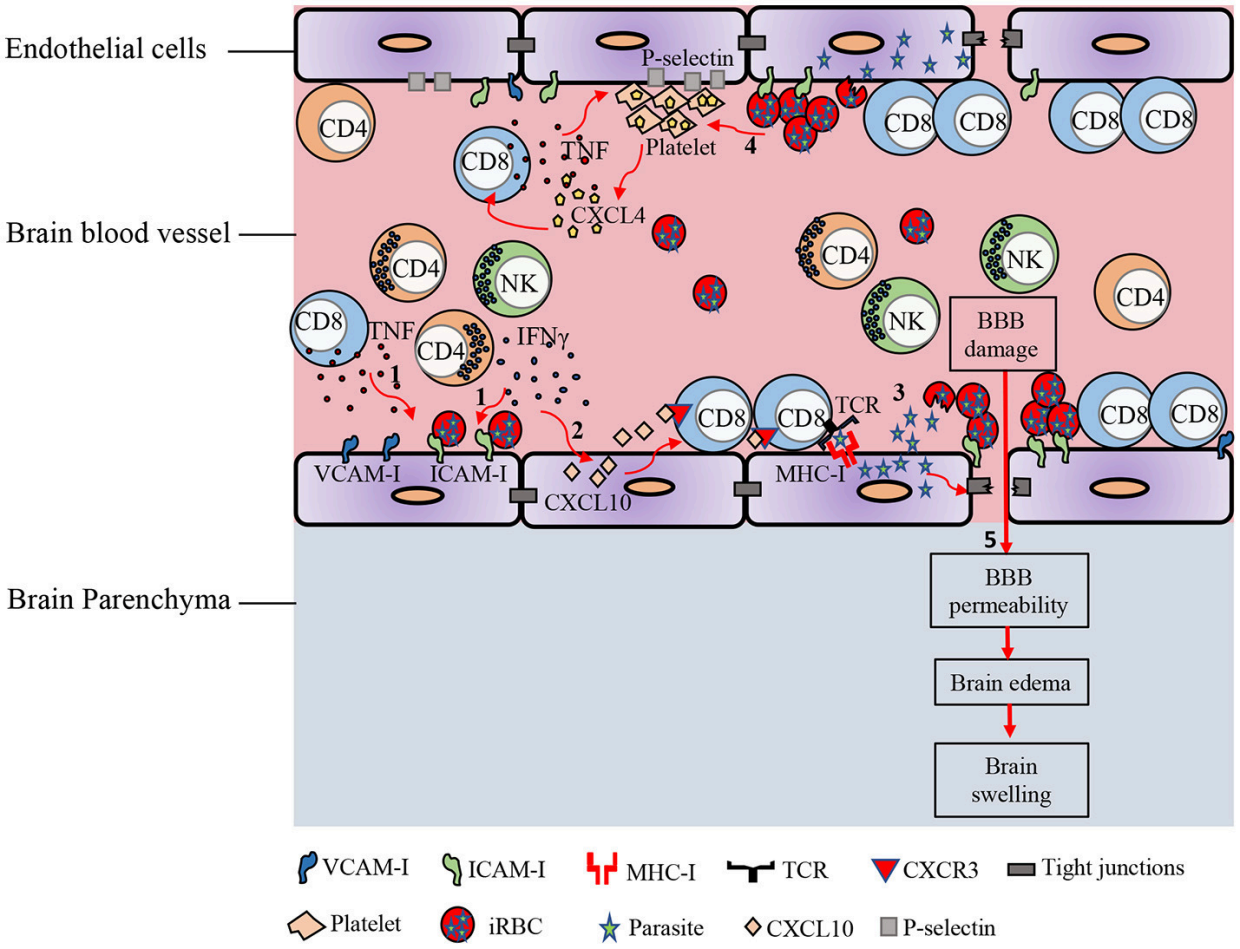
Immunopathology of ECM. In response to *Plasmodium* blood-stage infection, pro-inflammatory cytokines such as IFN-γ and TNF are produced by various immune cells. **(1)** These cytokines promote the up-regulation of adhesion molecules on brain endothelial cells. iRBCs sequester in the cerebral microvasculature and bind to receptors on the endothelial cells such as ICAM-1. **(2)** IFN-γ induces the expression of CXCL10 by brain endothelial cells. IFN-γ-induced CXCL10 enhances the adhesion of T cells to cerebrovascular endothelium and prevents their detachment from the brain vasculature. **(3)** Parasite antigens can be transferred from the iRBC into the endothelial cell. This interaction leads to opening of the intercellular tight junctions. Brain endothelial cells can phagocytize parasite antigens and cross-present them to CD8^+^ T lymphocytes. **(4)** iRBC can also directly activate platelets and stimulate the release of CXCL4 early in the course of ECM. CXCL4 induces the production of TNF by T cells and macrophages, which then causes more platelets to adhere to the endothelium. Enhanced expression of CXCR3-binding chemokines such as CXCL9, CXCL10, and CXCL4 in the brains of PbA-infected mice induces T cell migration to the brain in the late stage of ECM. **(5)** Accumulation of iRBCs and immune cells in cerebral vessels leads to vascular obstruction, increased pressure within the brain blood vessels, and increased permeability of the BBB, facilitating edema, and brain swelling.

Mouse models of ECM have been heavily criticized in the past for their lack of relevance to human CM, but recent studies (41, 50, 52, 53), suggest CM and ECM may be more similar than previously appreciated. Accumulation of iRBC and platelets in the brain, breakdown of the BBB, and swelling of the brain and its subsequent lethal consequences, all appear to be common attributes of both species. Recent studies in mice suggest techniques available to assess CD8^+^ T cell accumulation in human brains are inefficient and struggle to detect the relatively small number of infiltrating CD8^+^ T cells in histological sections (41), thus leaving this component of murine pathology potentially still relevant to human disease. A current simplified model of the pathogenesis of cerebral malaria suggests several important factors interplay to cause lethality (Figure 1). The primary requirement is sequestration of parasites in the brain. This is mediated by direct binding of iRBC to brain endothelium or through such interactions mediated by accumulating platelets. These iRBC-endothelial cell interaction leads to breakdown of the control of solute movement across the BBB, particularly within the post-capillary venules. This process, at least in mice, is amplified by CD8^+^ T cell recognition of parasite antigen on endothelial cells, possibly affecting the endothelial cells themselves or simply increasing cellular accumulation within the vessels. The accumulation of these and other leukocytes as well as iRBC within post-capillary venules increases blood pressure within the brain, further driving edema and ultimately leading to sufficient brain swelling to cause lethal herniation. While some direct killing of endothelial cells may be mediated by CD8^+^ T cells, causing overt bleeding, the primary mode of damage relates to increased permeability of the BBB, and increased pressure within the brain blood vessels, facilitating edema. Deciphering the precise mechanisms underlying the pathogenesis of CM is required for developing therapeutic approaches that can act to reduce or prevent death. Utilizing several different investigative approaches has improved our understanding of the cellular and molecular mechanisms underlying this disease. Use of new imaging methods and elucidation of the mechanistic basis of ECM in murine models may help better understand the spectrum of human disease and facilitate approaches for prevention of CM in humans.

## Author contributions

NG wrote the paper. SM and WH contributed to writing and critically revised the paper.

### Conflict of interest statement

The authors declare that the research was conducted in the absence of any commercial or financial relationships that could be construed as a potential conflict of interest.
